# Significant Nutritional Gaps in Tibetan Adults Living in Agricultural Counties Along Yarlung Zangbo River

**DOI:** 10.3389/fnut.2022.845026

**Published:** 2022-04-08

**Authors:** Chen-ni Zhou, Mo Li, Ran Xiao, Fang-jie Zhao, Fu-suo Zhang

**Affiliations:** ^1^Key Laboratory of Plant-Soil Interactions, Ministry of Education, College of Resources and Environmental Sciences, National Academy of Agriculture Green Development, China Agricultural University, Beijing, China; ^2^Key Laboratory of Alpine Vegetation Ecological Security in Tibet, Institute of Tibet Plateau Ecology, Tibet Agricultural and Animal Husbandry University, Nyingchi, China; ^3^Interdisciplinary Research Center for Agriculture Green Development in Yangtze River Basin, College of Resources and Environment, Southwest University, Chongqing, China; ^4^College of Resources and Environmental Science, Nanjing Agricultural University, Nanjing, China

**Keywords:** nutrient intakes, food sources, nutritional assessment, Tibetan adult population, Tibet Autonomous Region

## Abstract

**Background:**

Dietary intake and nutritional assessing data from a representative sample of adult population living in an agricultural zone on Tibet Plateau are still lacking nowadays. This study aimed to assess the daily dietary intakes and respective food sources in 552 local residents (≥ 18 years old, 277 men and 275 women) living in 14 agricultural counties along the Yarlung Zangbo River on Tibet Plateau.

**Methods:**

Food consumption data were collected using a validated cultural-specific food frequency questionnaire that contained all local Tibetan foods and analyzed with three fixed factors: gender, age, and region. Nutrient intakes were calculated using Chinese food composition tables. Nutritional gaps and the percentages of participants who had inadequate and excessive nutrient intakes were calculated by estimated average requirement (EAR) cut-point methods.

**Results:**

Compared with the dietary reference intakes, 68.4% of nutrient intakes were inadequate. Fiber, Ca, I, Zn, Se, and vitamin (Va, Vc, and folic acid) intakes appeared to be particularly deficient. The dietary energy intake was 7838.8 ± 537.1 KJ/d, with 78 and 84% of EAR values for men and women, respectively. The dietary intakes of most nutrients were below the estimated energy requirement/EAR or adequate intake values, while more than 70% of the participants had excessive intake of carbohydrate, especially the elderly (aged ≥ 51 years). The nutritional gap of Cu was more than 300%. Almost 100% of the participants was vulnerable to fiber, Se, and Va shortfalls due to the deficiency in sole food sources. The top five food sources of Se intake were highland barley (34.2%), meat (13%), rice (12.4%), eggs (12.2%), and cultural-specific beverages (7.8%). Eggs (42.1%), tubers (62.2%), vegetables (66.4%), and highland barley (49.7%) were the first contributors of Va, Ve, Vc, and folic acid, respectively.

**Conclusion:**

The dietary intake of a large sample of Tibetan adult population living in agricultural counties of Tibetan Autonomous Region is alarmingly insufficient. Gender inequality is common, and regional difference is widespread due to rapid urbanization. Young Tibetan adults aged 18–30 years are particularly vulnerable to micronutrient shortfalls and currently facing the risk of nutrition-insecurity-related dietary inadequacy. The respondents who belong to the elderly category (≥51 years of age) are facing the risk of “double burden of malnutrition” characterized by the coexistence of undernutrition, including micronutrient deficiencies and overweight or obesity.

## Introduction

A balanced diet that provides sufficient energy and essential nutrients is vital for protecting body health and preventing disease (1–3). Optimal nutritional intake is also essential for supporting metabolism and daily activity (4). Nutrient deficiencies or excesses play a role in the etiology of numerous diseases throughout the world (5–7). According to FAO, “food security” exists when all people, at all times, have physical, social, and economic access to sufficient safe and nutritious food that meets their dietary needs and food preferences for an active and healthy life (8). However, the term “food insecurity” was introduced by Campbell in 1990 (9) to indicate a limited availability of nutritionally adequate and safe foods or a limitation or uncertainty in the ability to acquire acceptable foods in socially acceptable manners (10, 11). In 2017, approximately 10% of the world population was severely food insecure compared with 8.4% in 2015 (12). Food and nutrition insecurity related to dietary inadequacy may lead to “double burden of malnutrition” (13), which is characterized by the coexistence of undernutrition, including micronutrient deficiencies (“hidden hunger”) and overweight or obesity (14, 15).

According to previous studies on the dietary intake of Tibetan residents, rural populations from the agricultural zones have a monotonous diet that is based on cereal products, with a very limited variety of food products (16, 17). Several studies focusing on anthropometric measurements and nutritional status assessment have observed clinical signs of malnutrition and concluded on a poor nutritional status of rural Tibetans, with a diet extremely low in vegetables and fruits but rich in cereals (18, 19). In the particular environment of rural Tibet Autonomous Region (T.A.R.), a rampant disease called Kashin–Beck disease (KBD) affects thousands of people (20–22). Although what exactly causes this disease is unclear, this endemic and chronic osteochondropathy may find its roots in multiple socioenvironmental causes, among which diet seems to play a part (23). More specifically, mineral deficiencies are thought to be involved in the high prevalence of rickets, and Se and I deficiencies are supposed to play a role in KBD (24, 25). Moreover, KBD is only encountered in the agricultural zone, and endemic areas are limited to poor, isolated, and rural communities (22, 25). Nutritional assessment data of adults in the agricultural zone are highly necessary; to the authors’ knowledge, such information from a representative sample of Tibetan adults is still lacking to date, even though few reports on the nutritional status of Tibetan mothers and children are available (18, 26, 27). Despite gender discrepancies and age and regional differences were emphasized in epidemiological studies of endemic diseases in Tibet, the dietary intakes of Tibetan residents of different genders, age categories, and regions remain unexplored. In this context, the present study aimed to (i) provide the latest estimates of the daily intake of dietary energy and 18 nutrients by Tibetan adult population aged ≥ 18 years living in agricultural counties of T.A.R.; (ii) obtain insights into the main food sources that contribute to these nutrient intakes; (iii) evaluate the nutrient intake adequacy among participants in accordance with the Chinese dietary reference intakes (DRIs). An enhanced understanding of dietary intakes and possible gender, age, and regional differences could be used to optimize the current interventions and design new nutritional interventions that aim to improve the dietary intakes of the local adult population of agricultural zones in T.A.R.

## Materials and Methods

### Study Design

**FIGURE 1:**
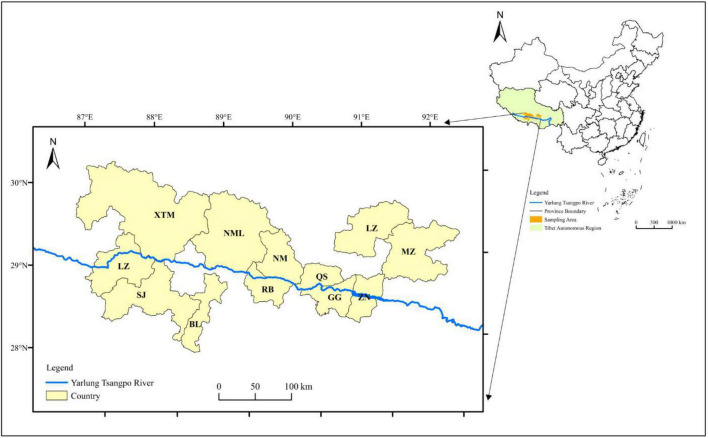
Distribution of sample counties.

Quantitative dietary data were collected from a cross-sectional survey carried out in August 2020 in 14 major agricultural counties (Medrogongkar, MZ; Chushur, QS; Nyemo, NM; Lhundup, LZH; Danang, ZN; Gonggar, GG; Sangzhuzi, SZZ; Namling, NML; Gyantse, JZ; Sakya, SJ; Lhatse, LZ; Thongmon, XTM; Panam, BL, and Rinpun, RB) along the Yarlung Zangbo River (Figure 1). A cultural-specific food frequency questionnaire that contained all local Tibetan foods, such as *tsampa* (flour obtained from roasted highland barley grains), Tibetan sweet tea (local black tea mixed with sweetened milk powder), yak buttered tea (salt cream tea), yak meat, and *chang* (wine made from highland barley in a unique local manner) was used to assess the types and frequency of food consumed (28). For each county, 20 urban residents and 20 rural residents were selected by stratified sampling by age and sex. All participants were divided into three age groups (L_18–30 years old; M_31–50 years old; H_ > 50 years old). A total of 552 participants were interviewed face-to-face by trained professional interviewers. The characteristics of subjects is shown in Table 1. We conducted a pre-survey to develop a draft food list in order to complete a cultural-specific FFQ in June 2020 in Lhundup County. Considering the relative singularity of dietary structure and ethnic uniqueness of Tibetan local residents, we first verified the validity of the FFQ by using a 2-consecutive-day 24 h dietary records (24-H DR) before being applied in the present study (Supplementary Figure 1). The FFQ was revised twice by local nutritional staff, and its re-tested results ensure that this cultural-specific FFQ can fit local daily food consumption patterns well (Supplementary Table 1). The final version of the FFQ included 165 food items with average standard portion size, proper frequency categories.

**TABLE 1:** Characteristics of subjects.

		Male (*n* = 277)		Female (*n* = 275)	
		Urban (*n* = 147)	Rural (*n* = 130)	Urban (*n* = 133)	Rural (*n* = 142)
Age (year)		43 ± 0.6	53 ± 0.8	40 ± 0.5	56 ± 0.8
Age groups (*n*)	18–30 years	40	34	31	44
	31–50 years	63	46	58	59
	≥ 51 years	44	50	44	39

### Nutrition Intake Database Building

The original collected data, including food items and food consumption data, were inputted into a computer in accordance with the standard portion size. Then, double data entry was performed. Any discrepancies were identified, checked against the original records, and corrected before data analysis. The daily intake amount of each food item per person was calculated. The Chinese Food Composition Table was used to estimate nutrient intakes (Standard Edition, version 6, 2019) (29). Nutrient intakes were calculated by multiplying the nutrient content by the daily intake of each food. The intake of each nutrient is the total amount of that nutrient provided by all foods. After checking for completeness, dietary energy (KJ/d) and 18 nutrients, including macronutrients such as protein (g/d), total fat (g/d), total carbohydrate (g/d), and fiber (g/d); minerals such as Ca (mg/d), phosphorus (mg/d), potassium (mg/d), sodium (mg/d), and magnesium (mg/d); micronutrients such as iron (mg/d), iodine (μg/d), zinc (mg/d), selenium (μg/d), and copper (mg/d); and vitamins such as vitamin A [μg retinol activity equivalents (RAEs)/d], vitamin E (mg/d), vitamin C (mg/d), and folic acid (μg/d), were analyzed. The total daily intake of each nutrient based on food consumption was calculated per person per day. Individual food items were coded and classified in 12 food groups on the basis of nutrient composition and culinary use. According to previous studies, the dietary structure of Tibetan residents is mainly characterized by the consumption of cereals (19, 26). Therefore, in the present study, cereal foods were divided the into four categories, including highland barley, rice, wheat, and tubers. The other eight food categories were meat, dairy products, eggs, legumes, vegetables, fruits, nuts, and cultural-specific beverages (such as yak buttered tea, Tibetan sweet tea, and *Chang*). The nutritional contribution rate of each food group was determined by dividing the total nutrient intake contributed from the specific food group by the total nutrient intake (for each separately) from all food groups, and then multiplying it by 100 (30).

### Nutritional Adequacy Assessment

The Chinese Dietary References Intakes (DRIs, 2013 version) (31) was used to evaluate the nutrient inadequacy and excessive intake of the participants. The estimated energy requirement (EER) and estimated average requirement (EAR) were used to evaluate energy as well as nutrients intakes. The EER value for the moderate level of physical activity was used to evaluate the dietary energy for urban adults, and the EER value for high level of physical activity was used to evaluate the dietary energy for rural adults. All nutrients with EARs were assessed using the EAR cut-point method (32). Nutrients with no EARs (such as vitamin E, sodium, and potassium) used the adequate intake (AI), because the EAR of these nutrients were not established in China yet. The proportion of individuals with excessive intakes was estimated using a tolerable upper intake level or upper limits per day (UL) as cut-off values. Acceptable macronutrient distribution ranges (AMDRs) were used to evaluate carbohydrate, protein, and fat intakes as a percentage of energy. The reference values of nutrient intakes based on Chinese DRIs are shown in Table 2. Nutritional gaps (%) = [(dietary intake − DRIs)/DRIs] × 100%.

**TABLE 2:** Reference values of nutrient intakes based on Chinese DRIs.

Nutrients	Age	Gender	EAR^a^	AI^a^	UL^a^
Energy/(kJ/d)	18^+^	M	10,880^b^/12,550^c^	–	–
		F	8,790^b^/10,040^c^	–	–
	50^+^	M	10,250^b^/11,720^c^	–	–
		F	8,580^b^/9,820^c^	–	–
Carbohydrate/(% EER)	–	–	120 g/50–65%^d^	–	–
Fat/(% EER)	–	–	20–30%^d^	–	–
Protein (% EER)		–	(male: 60 g, female: 50 g)/10–15%^d^	–	–
Fiber/(g/d)	–	–	25–30	–	–
Ca/(mg/d)	18^+^	–	650	–	2,000
	50^+^	–	800	–	
P/(mg/d)	–	–	600	–	3,500
K/(mg/d)	–	–	–	2000	–
Na/(mg/d)	18^+^	–	–	1500	–
	50^+^	–	–	1400	–
Mg/(mg/d)	–	–	280	–	–
Fe/(mg/d)	18^+^	M	9	–	42
		F	15	–	
	50^+^	–	9	–	
I/(μg/d)	–	–	85	–	600
Zn/(mg/d)	–	M	10.4	–	40
		F	6.1	–	
Se/(μg/d)	–	–	50	–	400
Cu/(mg/d)	–	–	0.6	–	8
Vitamin A/(μg/d)	–	–	560	–	3,000
Vitamin E/(mg/d)	–	–	–	14	700
Vitamin C/(mg/d)	–	–	85	–	2,000
Folic acid	–	–	320	–	1,000

*^a^Estimated Average Requirement (EAR) values, adequacy intake (AI), and Tolerable Upper Intake (ULs) for adults (18^+^ years old). ^b^Estimated energy requirement (EER) for moderate level of physical activity. ^c^Estimated energy requirement (EER) for high level of physical activity. ^d^Acceptable macronutrient distribution ranges (AMDRs) (31).*

### Statistical Analysis

Continuous variables were presented as mean (standard error, SE) and percentiles, while categorical variables were expressed as percentage. Shapiro–Wilk test was applied to detect possible non-normal distributions of the variables. When the statistical distribution was not normal, a logarithmic transformation of the variable was performed. The mean values of nutrient intakes were compared by ANOVA with three fixed factors: genders (two levels), age groups (three levels), and regions (two levels). *Post-hoc* tests were performed to compare the mean nutrient intakes among the three age groups. Data were analyzed using IBM SPSS 25.0 statistical software (IBM Corp., Armonk, NY, United States), and plotting was performed on Origin 2021b (Origin lab, Northampton, Massachusetts, United States). The level of significance was set at 0.05.

### Ethical Approval

This study was conducted according to the guidelines laid down in the Declaration of Helsinki and all procedures involving human subjects/patients were approved by the Chinese Center for Disease Control and Prevention (CDC) of Tibet Autonomous Region. All participants signed an informed consent before participation.

## Results

### Dietary Intakes of Energy and 18 Nutrients of Tibetan Adults Living in Agricultural Counties in Tibet Autonomous Region

**FIGURE 2:**
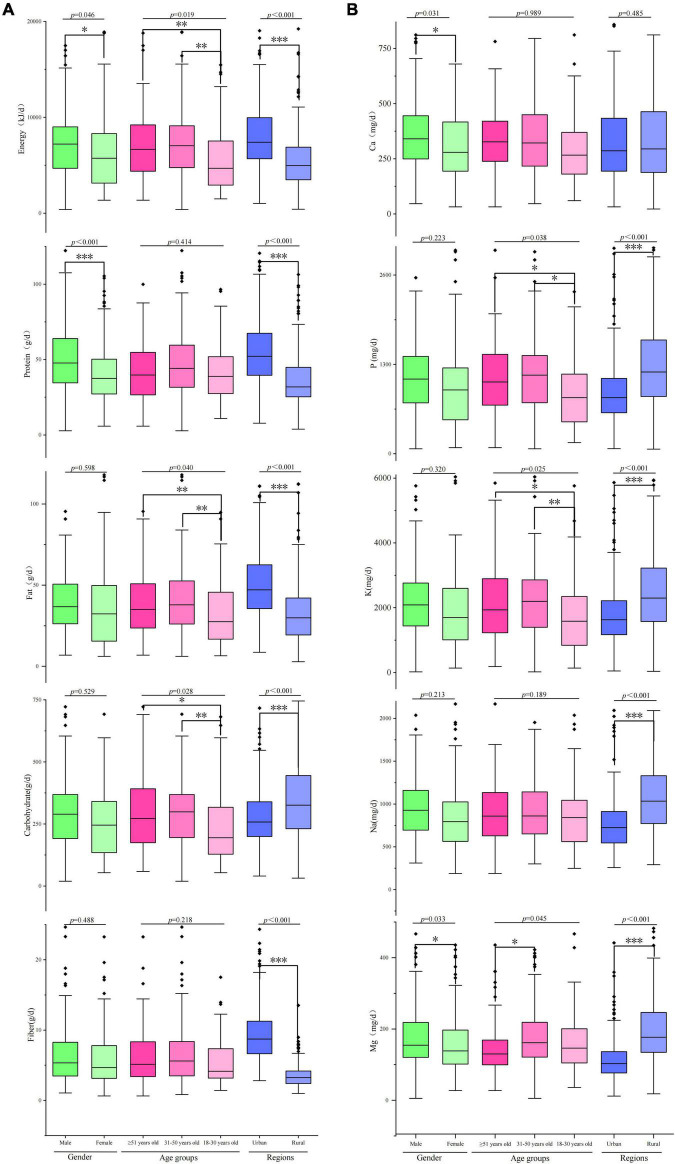
Dietary energy and macronutrient (carbohydrate, protein, fat, and fiber) and mineral (Ca, P, K, Na, and Mg) intakes of Tibetan adults living in agricultural counties of T.A.R. *p < 0.05; **p < 0.01; ***p < 0.001.

**FIGURE 3:**
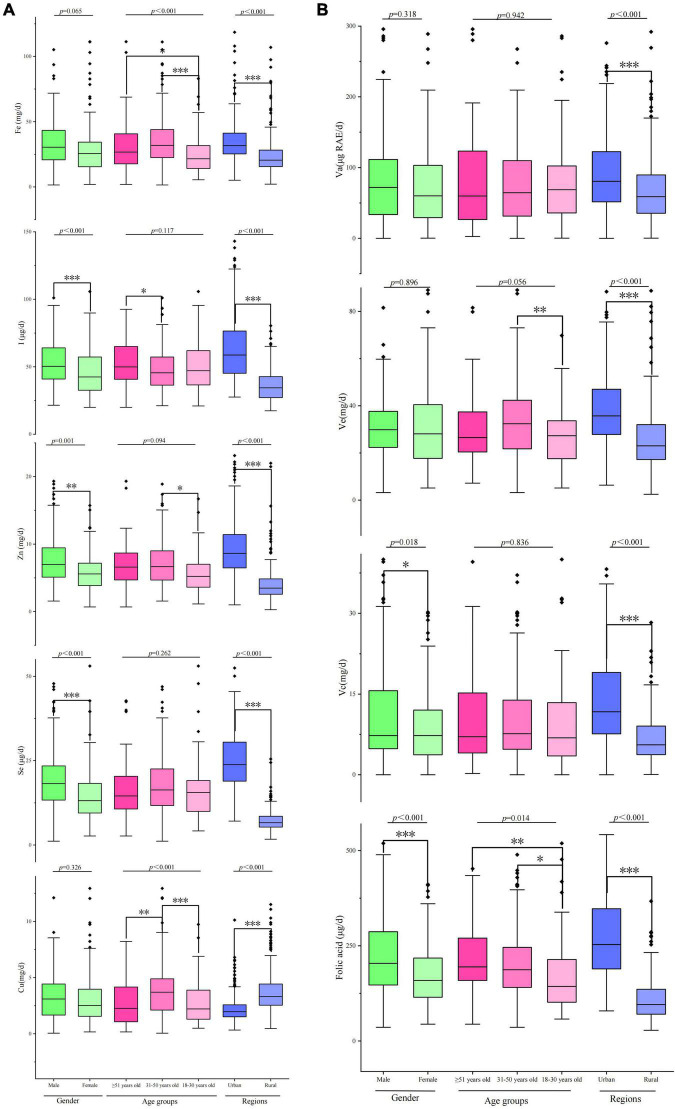
Micronutrient (Fe, I, Zn, Se, and Cu) and vitamin (Va, Ve, Vc, and folic acid) intakes of Tibetan adults living in agricultural counties of T.A.R. *p < 0.05; **p < 0.01; ***p < 0.001.

The dietary energy intake was 7838.8 ± 537.1 KJ/d, and the dietary intakes of protein, fat, and carbohydrate were 48.1 ± 2.4 KJ/d, 44.7 ± 4.2 KJ/d, and 321.3 ± 26.4 KJ/d, respectively. The energy intake of men (8480.1 ± 635.3 KJ/d) was significantly higher than that of women (7387.0 ± 680.4 KJ/d, *F* = 2.773, *p* = 0.046). The energy intake of Tibetan adults ≥ 51 years old was the highest among the three age groups (*F* = 4.013, *p* = 0.019). Tibetan adults living in urban areas (8857.8 ± 432.9 KJ/d) had larger energy intake than those living in rural areas (6129.5 ± 357.7 KJ/d, *F* = 23.602, *p* < 0.001). For macronutrients, no significant differences were found in the dietary intakes of fat, fiber, and carbohydrate between genders, except protein intake (*F* = 11.329, *p* < 0.001). However, the dietary intakes of fat and carbohydrate significantly differed among the three age groups. Except for carbohydrate (urban: 300.7 ± 13.9 g/d, rural: 389.7 ± 21.9 g/d, *p* < 0.001), the dietary intakes of the other three macronutrients (fat, protein, and fiber) in urban adults were significantly higher than those in rural adults (Figure 2). The mean mineral intakes of Ca, P, K, Na, and Mg of Tibetan adults living in agricultural counties were 380.1 ± 23.3, 1230.2 ± 89.4, 2366.3 ± 198.4, 988.9 ± 72.9, and 169.5 ± 8.2 mg/d, respectively. Men had higher daily intakes of minerals than women, and adults living in urban areas and young adults aged 18–30 years had low mineral intakes (Figure 2). The average daily intakes of micronutrients (Fe, I, Zn, Se, and Cu) were 31.4 ± 1.6 mg/d, 50.5 ± 2.3 μg/d, 7.2 ± 0.5 mg/d, 17.0 ± 0.7 μg/d, and 3.28 ± 0.15 mg/d, respectively. The I, Zn, and Se intakes of women was significantly lower than those of men (*p* < 0.001, *p* = 0.0017, and *p* < 0.001). Tibetan adult population aged 31–50 years had the largest intakes of most micronutrients, except for I (H > L > M). Urban adults had larger intakes of Fe, I, Zn, and Se than rural adults, except for Cu (urban: 2.49 ± 0.2 mg/d; rural: 4.33 ± 0.4 mg/d; *p* < 0.001, Figure 3). The mean intakes of Va, Ve, Vc, and folic acid were 82.7 ± 6.3 μg RAE/d, 35.3 ± 2.8 mg/d, 10.6 ± 0.9 mg/d, and 200.4 ± 7.8 μg/d, respectively. The Vc and folic acid intakes of women were significantly lower than those of men (*F* = 5.64, *p* = 0.018; *F* = 22.219, *p* < 0.001). The daily intakes of Va, Ve, and folic acid of young adults (18–30 years old) were the least among the three age groups. However, old adults (≥ 51 years old) had the most folic acid intake and the least Vc intake (Figure 3).

### Food Sources of Dietary Intakes of Tibetan Adults Living in Agricultural Counties in Tibet Autonomous Region

**FIGURE 4:**
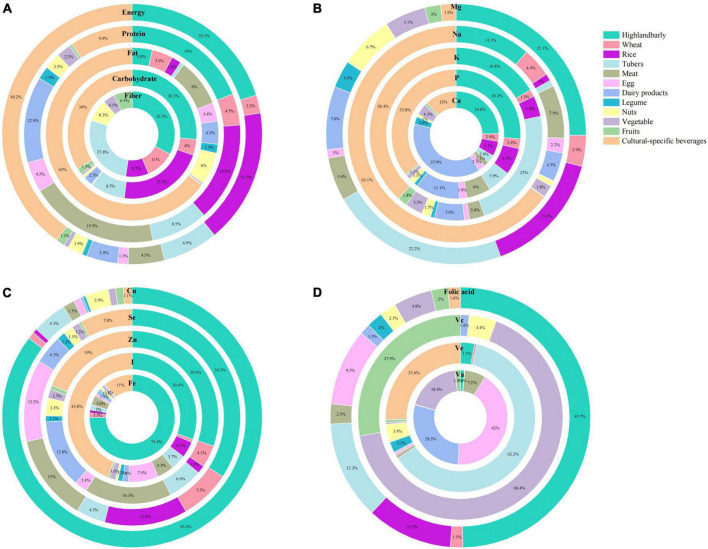
Mean contribution (%) of food groups to dietary intakes of **(A)** macronutrients (energy, protein, fat, carbohydrate, and fiber), **(B)** minerals (Ca, P, K, Na, and Mg), **(C)** micronutrients (Fe, I, Zn, Se, and Cu), and **(D)** vitamins (Va, Ve, Vc, and folic acid) among Tibetan adults living in agricultural counties of T.A.R.

The percentage contributions of nutrients to each food group are shown in Figure 4. The top five food sources of energy were cultural-specific beverages (40.2%), highland barley (20.2%), rice (16.7%), tubers (6.9%), and meat (3.9%). Meat (19.3%), highland barley (18.9%), rice (14.8%), dairy products (12.8%), and tubers (8.5%) were the top five contributors to protein. More than half of the total fat came solely from cultural-specific beverages (65%), followed by meat (8.9%), and nuts (6.1%). Thirty-four percent of carbohydrate came from cultural-specific beverages, followed by highland barley (26.3%) and rice (21.7%). Highland barley contributed the most to dietary fiber intake (32.7%), followed by tubers (27.8%) and wheat (11%). Dairy products and highland barley were the top two food sources of Ca, while cultural-specific beverages were the top food source of P and Na, followed by highland barley. For K, cultural-specific beverages and tubers were the top contributors. Highland barley and tubers contributed the most to Mg intake. For micronutrients, nearly 75% of Fe intake and 85% of Cu intake were supplied by highland barley. The top two contributors of Zn and I were cultural-specific beverages and highland barley. The top five food sources of Se intake were highland barley (34.2%), meat (13%), rice (12.4%), eggs (12.2%), and cultural-specific beverages (7.8%). For vitamins, eggs (42.1%), tubers (62.2%), vegetables (66.4%), and highland barley (49.7%) were the first contributors of Va, Ve, Vc, and folic acid, respectively.

### Assessment of Nutrient Intakes of Tibetan Adults Living in Agricultural Counties in Tibet Autonomous Region

The mean and SE of dietary intakes of energy and nutrients and the prevalence of deficient and excessive intakes among Tibetan adults living in agricultural counties in T.A.R. are shown in Tables 2–4. The dietary intakes of energy and most nutrients were all below the EER/EAR or AI, except for carbohydrate, P, Fe, Cu, and Ve. Carbohydrate and Cu intakes had the largest differences in terms of the intakes of excess nutrients and the reference values. However, they were still far lower than the tolerable upper intake levels. The nutritional gaps of dietary energy and DRIs between two genders were 22 and 16% (Table 2). Young adults (aged 18–30 years) and adult populations living in rural areas had a greater energy gap below EER at 45.3% (Table 3) and 51.2% (Table 4), respectively. In terms of macronutrients, residents in rural areas of Tibet are seriously inadequate in their intake of dietary fiber, with insufficient fiber intake in 100% male and 99.28% female participants. Obvious differences were observed in the nutritional gaps of protein and fat between urban and rural areas (protein: urban = 3.2%, rural = 37.3%; fat: urban = 1.1%, rural = 10.7%). 51.47% elderly adults (≥ 51 years old) had protein intake of lower than 25.8% of EAR values. However, their fat intake was 13.7% above the EAR values. More than 90% of Tibetan residents exhibited insufficient Ca intake, especially women and the elderly. In terms of the intake of micronutrients, Tibetan residents demonstrated a serious lack of I, Zn, and Se intake. In particular, the intake of Se is the most lacking. Almost all of the participants were at high risk of inadequate Se intake. In addition to the AI of vitamin E, the adult residents in Tibet’s agricultural areas are seriously deficient in the intake of the other three vitamins. Almost 100% of Tibetan adults’ intake of vitamins A and C was lower than the EAR value, and the nutritional gap was above 85%. Women, rural adults, and the young adults aged 18–30 years had the largest nutritional gaps of folic acid (94.28, 99.27, and 91.46% below EAR values, respectively).

**TABLE 3:** Nutrient gaps and prevalence of inadequate and excessive intake of nutrients by gender.

Nutrients	Male				Female			
	Mean (SE)	Nutritional Gaps (%)^#^	% Subjects		Mean (SE)	Nutritional Gaps (%)^#^	% Subjects	
						
			< EAR (%)^$^	> EAR (%)^&^			< EAR (%)^$^	> EAR (%)^&^
Energy/(kJ/d)^a^	8480 (635)	−22	82.84	17.16	7387 (680)	−16	77.85	22.15
Carbohydrate/(g/d)	333.8 (25.8)	178^b^	6.71^c^	68.65^c^	309.6 (28.4)b	158^b^	2.85^c^	79.28^c^
Fat/(%E)^c^	20.5 (0.8)	−18	48.51	2.98	18.5 (0.6)	−26	40	2.85
Protein/(g/d)	54.0 (2.9)	−10^b^	41.79^c^	26.12^c^	42.3 (1.9)	−15.4^b^	40.71^c^	32.14^c^
Fiber/(g/d)^c^	6.4 (0.4)	−78.7	100	−	6.0 (0.4)	−80	99.28	−
Ca/(mg/d)	426.1 (39.3)	−34.4^b^	90.29^b^	1.49^e^	333.3 (18.6)	−48.7^b^	95^b^	−
P/(mg/d)	1319.1 (100.1)	120^b^	15.67^b^	4.47^e^	1143.2 (103.5)	90.5^b^	29.28^b^	2.14^e^
K/(mg/d)^d^	2510.5 (190.5)	26	47.01	52.99	2230.9 (206.1)	11.5	60	40
Na/(mg/d)^d^	1047.7 (58.6)	−30	90.29	9.71	935.7 (67.5)	−37.6	91.43	8.57
Mg/(mg/d)^b^	182.0 (9.5)	−35	87.31	12.69	156.9 (7.1)	−43.9	90.71	9.29
Fe/(mg/d)	33.7 (1.6)	145.7^b^	−	27.61^e^	29.3 (1.7)	115.3^b^	14.28^b^	15.71^e^
I/(μg/d)^b^	54.4 (1.6)	−36	94.03	−	46.5 (1.5)	−45.3	96.42	−
Zn/(mg/d)^b^	8.1 (0.4)	−22.1	83.58	−	6.3 (0.4)	3.3	58.57	−
Se/(μg/d)^b^	19.3 (0.8)	−61.4	100	−	14.9 (0.7)	−58.5	99.28	−
Cu/(mg/d)	3.4 (0.2)	466.7^b^	5.22^b^	5.97^e^	3.2 (0.2)	433.3^b^	7.85^b^	4.28^e^
Vitamin A/(μg RAE/d)^b^	87.5 (6.1)	−84.4	100	−	78.4 (6.7)	−86	99.28	−
Vitamin E/(mg/d)^d^	35.5 (2.5)	153.6	6.71	−	35.0 (2.9)	150	12.85	−
Vitamin C/(mg/d)^b^	12.1 (1.1)	−85.8	99.25	−	9.1 (0.6)	−89.3	100	–
Folic acid ((μg/d)^b^	228.8 (9.8)	−28.5	77.61	−	172.9 (6.7)	−45.9	94.28	−

*^a^Compared with EER, ^b^Compared with EAR, ^c^Compared with AMDR, ^d^Compared with AI, ^e^Compared with UL. ^#^Nutritional gaps (%) = [(dietary intake−DRIs)/DRIs] × 100%. ^$^Percentage of adult population with nutrient intakes lower than EER/EAR/AI/AMDR. ^&^Percentage of adult population with nutrient intakes above EER/EAR/AI/AMDR/UL.*

**TABLE 4:** Nutrient gaps and prevalence of inadequate and excessive intake of nutrients by age group.

Nutrients	L (18–30 years old)				M (31–50 years old)				H (≥ 51 years old)			
	Mean (SE)	Nutritional gaps (%)^#^	% Subjects		Mean (SE)	Nutrient gaps (%)^#^	% Subjects		Mean(S.E.)	Nutritional gaps (%)^#^	% Subjects	
									
			< EAR (%)^[*dollar*]^	> EAR (%)^&^			< EAR (%)^[*dollar*]^	> EAR (%)^&^			< EAR (%)	> EAR (%)
Energy/(kJ/d)^a^	5951 (414)	−45.3	89.02	10.98	8562 (727)	−21.3	83.87	16.13	9129 (1203)	−10.9	80.88	19.12
Carbohydrate/(g/d)	243.8 (17.5)	103.2^b^	4.87^c^	70.73^c^	348.8 (30.3)	190.7^b^	4.83^c^	73.38^c^	365.2 (48.7)	204.3^b^	4.41^c^	79.41^c^
Fat/(%E)^c^	18.7 (0.6)	−6.3	47.56	6.09	20.9 (0.8)	−4.1	42.74	1.61	11.3 (0.3)	13.7	42.64	1.47
Protein/(g/d)	46.9 (3.7)	−21.8^b^	29.26^c^	41.46^c^	50.5 (2.6)	−15.8^b^	43.54^c^	24.19^c^	44.5 (3.1)	−25.8^b^	51.47^c^	23.52^c^
Fiber/(g/d)^c^	5.5 (0.4)	−81.7	98.78	−	6.6 (0.4)	−78	100	−	6.3 (0.5)	−79	100	−
Ca/(mg/d)	374.2 (59.6)	−42.4^b^	95.12^b^	2.43^e^	381.5 (21.5)	−33.6^b^	91.12^b^	−	378.9 (21.5)	−52.6^b^	95.58^b^	−
P/(mg/d)	949.3 (82.7)	58.2^b^	41.46^b^	2.43^e^	1331.4 (111.1)	121.9^b^	16.93^b^	3.22^e^	1380.4 (179.3)	130.1^b^	20.58^b^	4.41^e^
K/(mg/d)^d^	1787.8 (131.7)	−10.6	65.85	34.14	2614.3 (221.1)	30.7	45.96	54.04	2617.0 (356.2)	30.9	52.94	47.06
Na/(mg/d)^d^	868.6 (47.1)	−42.1	92.68	7.32	1025.6 (66.9)	−31.6	90.32	9.68	1073.5 (116.6)	−30.5	85.29	14.71
Mg/(mg/d)^b^	167.6 (11.2)	−40.1	90.24	9.76	182.9 (9.1)	−34.6	87.09	12.91	146.2 (10.1)	−47.8	91.17	8.82
Fe/(mg/d)	24.6 (1.6)	64^b^	30.48^b^	8.53^e^	36.1 (1.9)	140.7^b^	11.29^b^	29.83^e^	31.1 (2.4)	245.6^b^	4.41^b^	22.05^e^
I/(μg/d)^b^	51.5 (2.2)	−39.4	90.58	−	48.1 (1.5)	−43.4	97.58	−	53.6 (2.2)	−36.9	92.64	−
Zn/(mg/d)^b^	6.2 (0.5)	−40.4	91.46	−	7.6 (0.4)	−53.8	83.06	−	7.5 (0.6)	−27.9	86.76	–
Se/(μg/d)^b^	16.2 (0.9)	−67.6	98.78	−	17.9 (0.8)	−64.2	100	−	16.4 (1.0)	−67.2	100	–
Cu/(mg/d)	2.7 (0.2)	350^b^	3.65^b^	2.43^e^	3.9 (0.2)	550^b^	5.64^b^	8.06^e^	2.9 (0.3)	383.3^b^	11.76^b^	2.94^e^
Vitamin A/(μg RAE/d)^b^	81.3 (7.4)	−85.5	100	−	84.7 (7.3)	−84.9	99.19	−	81.8 (8.6)	−85.4	100	–
Vitamin E/(mg/d)^d^	28.3 (1.7)	102.1	17.07	−	38.4 (2.9)	174.3	6.45	−	37.9 (5.1)	170.7	7.35	−
Vitamin C/(mg/d)^b^	10.9 (1.5)	−87.2	98.78	−	10.7 (0.8)	−87.4	100	−	9.9 (0.9)	−88.4	100	−
Folic acid ((μg/d)^b^	174.2 (11.9)	−45.6	91.46	−	206.9 (8.9)	−35.3	83.87	−	219.5 (11.5)	−31.4	83.82	−

*^a^Compared with EER, ^b^Compared with EAR, ^c^Compared with AMDR, ^d^Compared with AI, ^e^Compared with UL. ^#^Nutritional gaps (%) = [(dietary intake−DRIs)/DRIs] × 100%. ^$^Percentage of adult population with nutrient intakes lower than EER/EAR/AI/AMDR. ^&^Percentage of adult population with nutrient intakes above EER/EAR/AI/AMDR/UL.*

## Discussion

### Nutrient Intakes of Tibetan Adults Living in Agricultural Counties of Tibet Autonomous Region

This cross-section survey provided detailed data on the daily nutrient intakes and nutritional status of local Tibetan adults living in agricultural countries along the Yarlung Zangbo River. The results showed that the nutrient intake of Tibetan adults mainly depends on the unique local foods and not nutritionally balanced. Overall, almost 70% of the nutrients investigated in this study were inadequate. Compared with the DRIs, vitamins (Va, Vc), fiber and micronutrients (I, Zn, and Se) intakes are appeared to be particularly deficient.

The present study found that 20% of the respondents in the study area had a deficit in dietary energy intake. As early as the 1960s, researchers had found that high altitude Himalayan residents live at latitudes considered tropical, yet inhabit substantially cooler thermal environments than the surrounding lowlands and may have higher BMRs (base metabolic rates) than predicted on the basis of Euroamerican equations (33). Therefore, there is a gap between BMRs requirement and actual energy intake. It should be noted that the deficiency of energy was prevalent among young Tibetan adults (aged 18–30 years, as high as 45.3%, Table 4) and rural residents (as high as 51.2%, Table 5), which was inconsistent with other studies found insufficient energy intake was widespread among the old adults (30, 34). Interestingly, the present study found that the dietary energy deficit of the elderly (≥ 51 years old) is only about 10% (Table 4), which is due to the fact that residents of this age group mostly followed the “local traditional diet” characterized by a high intake of Tsampa (roasted highland barley flour), cultural-specific beverages (Tibetan sweet tea and yak buttered tea), potato, and yak beef (28). Dietary energy is essential for physiological processes, including locomotion, thermoregulation, reproduction, and growth (35). Prolonged periods of energy deficit can negatively impact health and performance, because low energy availability can alter endocrine signaling from the central nervous system (i.e., decreased release of gonadotropin releasing hormone to suppress reproductive function) in response to acute changes in cellular fuel oxidation and peripheral hormones (36), muscle growth and repair, hemoglobin synthesis, bone formation and repair, immune function, and cardiovascular function were affected significantly (37).

**TABLE 5:** Nutrient gaps and prevalence of inadequate and excessive intake of nutrients by region.

Nutrients	Urban				Rural			
	Mean (SE)	Nutritional Gaps (%)^#^	% Subjects		Mean (SE)	Nutritional Gaps (%)^#^	% Subjects	
						
			< EAR (%)^[*dollar*]^	> EAR (%)^&^			< EAR (%)^[*dollar*]^	> EAR (%)^&^
Energy/(kJ/d)^a^	8857 (432)	−18.6	82.48	17.52	6129 (357)	−51.2	94.16	5.84
Carbohydrate/(g/d)	300.7 (14.0)	150.6^b^	7.66^c^	9.85^c^	389.7 (21.9)	224.7^b^	3.28^c^	93.79^c^
Fat/(%E)^c^	23.9 (0.3)	−1.1	12.77	3.64	14.3 (0.06)	−10.7	18.97	0.72
Protein/(g/d)	58.1 (1.7)	−3.2^b^	32.84^c^	29.56^c^	37.6 (1.3)	−37.3	41.24	28.83
Fiber/(g/d)^c^	9.9 (0.5)	−67	98.54	0.72	3.7 (0.2)	−87.7^b^	99.63^c^	0.36^c^
Ca/(mg/d)	360.5 (18.8)	−45.5^b^	90.51^b^	0.72^e^	380.6 (21.9)	−33.8^b^	87.22^b^	0.72^e^
P/(mg/d)	963.9 (44.8)	60.7^b^	25.91^b^	1.45^e^	1320.4 (79.8)	120.1^b^	14.59^b^	4.74^e^
K/(mg/d)^d^	2001.3 (105.6)	0.06	67.15	32.85	2829.4 (168.5)	41.5	42.33	57.66
Na/(mg/d)^d^	782.6 (24.6)	47.8	96.35	3.65	1178.4 (51.3)	−21.4	83.57	16.43
Mg/(mg/d)^b^	119.5 (7.5)	−57.3	97.81	2.19	220.5 (17.9)	−21.3	85.40	14.60
Fe/(mg/d)	37.7 (1.8)	151.3^b^	4.01^b^	22.99^e^	25.3 (1.5)	114.4^b^	22.26^b^	7.29^e^
I/(μg/d)^b^	63.9 (1.5)	−24.8	80.65	−	36.4 (0.7)	−57.1	100	−
Zn/(mg/d)	10.1 (0.5)	−2.8^b^	66.05^b^	0.72^e^	4.3 (0.2)	−58.7^b^	96.71^b^	0.36^e^
Se/(μg/d)^b^	26.4 (1.2)	−47.2	97.08	−	7.7 (0.5)	−84.6	99.63	−
Cu/(mg/d)	2.5 (0.2)	316.7^b^	0.72^b^	1.09^e^	4.3 (0.4)	616.7^b^	0.72^b^	6.20^e^
Vitamin A/(μg RAE/d)^b^	94.5 (3.7)	−83.1	100	−	70.3 (3.2)	−87.4	100	−
Vitamin E/(mg/d)^d^	42.4 (1.8)	202.8	1.45	−	28.1 (1.3)	100.7	12.04	−
Vitamin C/(mg/d)^b^	14.3 (0.6)	−83.2	99.63	−	6.9 (0.3)	91.9	100	−
Folic acid/(μg/d)	286.5 (12.5)	10.5^b^	69.34^b^	0.36^e^	114.3 (7.1)	64.3^b^	99.27^b^	0.36^e^

*^a^Compared with EER, ^b^Compared with EAR, ^c^Compared with AMDR, ^d^Compared with AI, ^e^Compared with UL. ^#^Nutritional gaps (%) = [(dietary intake-DRIs)/DRIs] × 100%. ^$^Percentage of adult population with nutrient intakes lower than EER/EAR/AI/AMDR. ^&^Percentage of adult population with nutrient intakes above EER/EAR/AI/AMDR/UL.*

We also found that among the three major energy-supplying nutrients, fiber intake of the respondents was seriously insufficient. The surveyed Tibetan residents only consumed about 20% of the DRIs on average (Tables 3, 4), and even urban residents only consumed about 30% of the DRIs (Table 5). Dietary fiber intake in Tibet was currently estimated to be less than 10 g per person per day (Figure 2). However, nutritionists recommend intakes of 25–30 g per person per day (Table 2). Dietary fibers are an important part of the daily diet (38). Lack of fibers in the diet can lead to the pathological changes in organs and systems of human body and is associated with gastrointestinal diseases, cardiovascular diseases, and metabolic diseases, including obesity and diabetes (39, 40). Half of those fibers should come from the cereal bran and the rest from fruits and vegetables (41), which were seriously lacking in Tibet. The development of fiber-enriched foods would help Tibetans to meet such recommendations.

Thanks to the iodized salt program started in 1995 by the Chinese government, the majority of the rural families have access to this essential element for the growth that is iodine (42). However, the present study also found there were a iodine intake gap up to 40% (Tables 3, 4), which was consistent with the iodized salt coverage in Tibet (30%) (43). Iodine is an essential trace element for normal growth and brain development in human beings (44). Insufficient iodine intake can have serious consequences-including mental retardation of infants, miscarriages, other pregnancy complications, and infertility-collectively known as iodine-deficiency disorders (IDD) (44). Limited funding, the dispersed population, inadequate transport, and the exchanging of salt for grain in some communities made salt iodization impossible to sustain as a strategy to prevent IDD in Tibet (45).

The usual intake of Zinc calculated in this study provides only 40% of the Chinese RNI, which was close to that of young Children Living in Rural Areas of Tibet Autonomous Region (36%) (27). It is highly probable that Tibetan people, with high consumption of tsampa, richer in phytic acid than wheat flour and rice, have higher phytate intakes and present even more unfavorable molar ratios of phytate-to-zinc (46). An ideal solution to the problem of low intakes in zinc would be a higher consumption of meat and fish. However, fishes are scarce in many rural areas and are never consumed by local people for cultural reasons (47).

The usual selenium intake fulfilled less than 45% of the Chinese RNI (Tables 3, 4), selenium deficiency is more serious in rural (15% of the Chinese RNI) (Table 5), and it is probably overestimated. Many vegetables and culture-specific foods consumed by Tibetans are produced locally but have not been analyzed for their selenium content and some previous studies have revealed significant discrepancies between the food composition in Tibet and the Chinese FCTs (48). Selenium deficiency has been clearly observed among the Tibetan populations living in areas endemic for the Kashin-Beck disease (22, 23). If the imbalances in the other trace elements could be relatively easily solved through food diversification (27), the selenium issue is much more challenging for its deficiency is rooted in a serious deficiency of the soil in Tibet (49). Although the beneficial effects of the consumption of selenium-enriched salt on the prevalence of Kashin-Beck disease in Tibet have been observed, it is clearly not a long-term sustainable solution (50), and its implementation on a large scale would be difficult. Selenium-supplemented fertilizer is perhaps the most appropriate solution to the selenium issue in T.A.R., which has been shown to increase the average Se content in total diets (51) and have benefits on the crop yields and animal nutrition (52).

Vitamin A and vitamin C intakes were significantly deficient in the respondents, which fulfilled less than 45% of the Chinese EARs. From a study on the estimation of daily vitamin C intake in Chinese adults by using cross-sectional data from the 2015 China Nutritional Transition Cohort Study, dietary vitamin C intake was found to be inadequate in Chinese adult population (78.1 mg/d) (53). In the present study, the daily vitamin C intake of Tibetan adults was far below the average level of Chinese adult population. This finding is consistent with the result of a cross-sectional survey of rural Tibetan mothers (18), which indicated that participants had extremely insufficient intake of light vegetables, dark vegetables, and fruits, which were the top three food sources of vitamin C (53). Vitamin A is essential for growth and immune function, and deficiencies make people susceptible to infections and death due to depressed immune function (54, 55), it is also vital for vision and is especially important in pregnancy, as it contributes to organogenesis during early fetal development (56). Strategies to address vitamin A and vitamin C deficiency include supplementation, dietary diversification, and food fortification (57). However, resource scarcity, ineffective program delivery as well as inadequate enforcement of regulations in Tibet were response for the failing of utilizing the right mix of these strategies.

### Food Sources of Tibetan Adults Living in Agricultural Counties of Tibet Autonomous Region

The contribution of different food groups to nutrient intake is highly dependent on the dietary habits of the study population (58). Highland barley and cultural-specific beverages were the main contributors for energy and nutrients for local Tibetan residents (Figure 4). The extent to which their unique diet contributes to the nutritional health of the local population was clearly visible.

Highland barley is the dominant staple food on the Tibet Plateau regardless of the planting area (54.67%) or yields (70.25%) (59). Moreover, it has been an important food type for local residents to adapt to climate change for a long period (19). A flour made of roasted barley grains, called “*Tsampa*,” is the most important food in the Tibetan diet and eaten mixed with yak butter tea and without cooking. Tea is essential in the Tibetan diet and culture; it is made in a local cooking style. Due to its specific brewing method, it must be considered as an ingredient in its own (60). A whole tea brick (made of compressed black tea leaves) is generally brewed in several liters of boiling water for more than 1 h. The resultant liquid, a kind of concentrated brewed black tea added with salt and yak butter, is made into yak buttered tea, or added with milk powder, is made into Tibetan milk tea. The consumption frequencies of yak butter tea and Tibetan milk tea were approximately more than once per day (26), and their intakes were surprisingly large as most Tibetans have them instead of drinking water. According to the national FCTs, the energy of yak butter was extremely high (2958 KJ/100 g). Thus, cultural-specific beverages could provide large dietary energy.

In the current study, dairy products contributed most to the dietary intake of Ca (32%, Figure 4), comparable to the studies in Korea (29.6%) (61). On the contrary, drinking water was the main contributor of Ca intake in the general adult population in Shiraz, Iran (31). Though foods of animal origin, including poultry, meat, and fish, and protein-rich plant food products, such as nuts and legumes, are the main sources of dietary P (62), the total dietary intake of P was mostly contributed by cultural-specific beverages in the present study. This result could be explained by the milk and dairy food groups contributing 27% of the dietary intake of P (63). In line with the Newcastle 85^+^ study (non-alcohol beverage accounts for 19% of K intake) (29), cultural-specific beverages were found to account for 57.19% of dietary K intake. A review of available literature revealed that vegetables were among the food groups that contributed most to the dietary intake of Mg (64, 65). However, locally produced highland barley and tubers were the main source of dietary intake of Mg in agricultural zones of T.A.R.

Although meat and its products have been identified as the major source of dietary intake of Zn in many countries (65, 66), in some other regions, other food groups, such as dried and smoked seafood, grains, and cereals, contributed most to the intake of Zn (67, 68). In the present study, cultural-specific beverages and highland barley contributed most to the dietary intake of Zn. The Zn content in foods has been suggested not to be dependent on food origin or affected by cooking process (61). Therefore, the transformation of nutrients in the processing of Tibetan traditional foods is worth considering.

Drinking water has been shown to have a considerable contribution to the dietary intake of Cu in other regions (69), not in agreement with the results of the present study. Among the study food groups, highland barley contributed 83.53% of the dietary exposure of Cu. An interesting finding was cultural-specific beverages (42.4%) and condiments (40.5%) were the main source of Na intake. It is quite different from the results of previous studies, which indicated that processed foods were the major contributors of Na intake, because Na is inherent in foods; it is added in food processing (contributed 56.4%), and salt is added at the table or during home cooking (leading to 43.6% of dietary exposure to Na) (70, 71). A cross-section study that aimed to estimate the dietary vitamin C intake in Chinese adults has shown that the top four food sources of vitamin C were light vegetables, dark vegetables, fruits, and tubers, contributing to a combined 97.3% of total daily dietary vitamin C intake in the study population (53). In the current study, vegetable (66.4%), fruits (27.9%), and nuts (4.35%) were the top three food sources of vitamin C intake in Tibetan adults.

### Strengths and Limitations

To the authors’ knowledge, this study was the first report to investigate the dietary intakes on the Tibet Plateau, with a focus on agricultural region and involving a substantial number of participants. The study sample was representative for the adult population of Tibet, as participants of all age groups (from 18 years old to more than 51 years old) were included, and men, women, and rural and urban residents were more or less equally represented. This strength enables recommendations tailored to the needs of every adult individual. Moreover, dietary intakes of energy, all macronutrients (carbohydrate, protein, fat, and fiber), five mineral elements (Ca, P, K, Na, and Mg), five micronutrients (Fe, I, Zn, Se, and Cu) and four vitamins (Va, Ve, Vc, and folic acid) were estimated, thus providing a clear indication of the overall dietary intake of Tibetan adult population living in agricultural counties along the Yarlung Zangbo River.

However, the present study also has limitations. First, although some nutrition studies about Tibetan young children (27) and Tibetan rural mothers (18, 26) also used FCTs, together with individual surveys to estimate the nutritional status of local Tibetan people, other studies indicated that FCT-based nutrition surveys are not reliable enough to estimate the deficiency and toxicity (58). A survey of minerals and trace elements in traditional foods of rural areas of Lhasa Prefecture revealed significant discrepancies between the food composition in Tibet and the Chinese FCTs (60). Another measurement of the mineral content of eight Tibetan staple foods showed that more than 50–60% of *p*-values < 0.05 were highlighted (72). All these results suggested the inappropriateness of using FCTs as a reference for nutrition surveys in rural Tibet and emphasized the importance of food analysis for nutritional assessment in T.A.R. In brief, in conducting a nutrition survey in an area as remote as T.A.R, the use of FCTs is potentially inadequate to assess accurate nutrient intakes (61, 73), especially for trace elements, which are extremely sensitive for the low levels of DRIs (74, 75). The present study referred to the newest version of FCTs (sixth version, 2019) (29), which included most of traditional Tibetan foods, such as highland barley (food code: 014202), Tibetan eggs (food code: 111110), yak meat (food code: 082120), yak butter (food code: 105009), yak butter tea (food code: 105010), and milk dreg (food code: 109009). However, Tibet is a vast region with great differences in geographic units and crop production. All local produced traditional foods should be carefully analyzed for more accurate nutritional assessment. Further research needs to be considered based on this information, with a focus on the nutrition components of traditional Tibetan foods.

Secondly, data on sociodemographic characteristics, such as education, occupation, behavior, and life factors, were not collected, and they may also be associated with dietary intake (58, 73, 76, 77). In the present study, differences in nutrient intake were analyzed by gender, age, and region. However, the interactions were not considered and the sample size was needed be expanded, the results can only represent the situation in the research area, not the situation in Tibet as a whole. Furthermore, dietary intakes were not adjusted with energy intake, which may result in different findings, especially for gender and age comparisons.

Lastly, previous studies demonstrated culturally driven dietary seasonality among Tibetan Nomads, with low basal metabolic rate and low caloric intake in summer (33). In the present study, the dietary seasonal variation was not taken into consideration. Further studies should be conducted to more accurately estimate long-term usual intake on the basis of different seasons.

## Conclusion

Published data on the dietary intakes of Tibetan adult population are very limited. The present study provided a clear indication of the overall dietary intake of Tibetan adult population living in agricultural counties along the Yarlung Zangbo River. Overall, the dietary intake of a large sample of Tibetan adult population was alarmingly insufficient, especially for fiber, micronutrients, and vitamins. Gender inequality was common, and a significant difference was found in energy, protein, Ca, I, Zn, Se, vitamin C, and folic acid intakes between men and women. Regional differences were also widespread due to rapid urbanization. Most of nutrient intakes of urban adults were significantly higher than those of rural populations, while the intakes of carbohydrate and minerals (Ca, P, K, Na, and Mg) of rural adults were 29.6, 5.6, 37.1, 41.5, 50.6, and 84.8% more than those of urban adults, respectively. As for different age groups, young Tibetan adults aged 18–30 years old were particularly vulnerable to micronutrient shortfalls and currently face the risk of nutrition-insecurity-related dietary inadequacy. Meanwhile, the respondents who belong to the elderly category (≥ 51 years of age) face the risk of “double burden of malnutrition” characterized by the coexistence of undernutrition, including micronutrient deficiencies and overweight or obesity. In accordance with the unique food culture in Tibet, the traditional food groups (highland barley and cultural-specific beverages) contributed greatly to the nutrition of local residents. These findings emphasized the importance of nutritional interventions targeting all Tibetan adult populations living in agricultural zones on the Tibetan Plateau. Policy makers should focus on evaluating and modifying the current nutritional interventions and developing new ones, with a particular focus not only on the population living in endemic area but also on ordinary residents. Tailored interventions are needed to achieve and maintain good nutritional health throughout the whole lifespan and reduce the prevalence of food insecurity and “double burden of malnutrition.”

## Data Availability Statement

The original contributions presented in the study are included in the article/Supplementary Material, further inquiries can be directed to the corresponding author/s.

## Ethics Statement

The studies involving human participants were reviewed and approved by the Chinese Center for Disease Control and Prevention (CDC) of Tibet Autonomous Region. The patients/participants provided their written informed consent to participate in this study.

## Author Contributions

F-SZ: supervision. ML: data collection and survey. RX: data analysis. C-NZ: data collection, survey, data analysis, and manuscript writing. F-JZ: supervision and data analysis. All authors contributed to the article and approved the submitted version.

## Conflict of Interest

The authors declare that the research was conducted in the absence of any commercial or financial relationships that could be construed as a potential conflict of interest.

## Publisher’s Note

All claims expressed in this article are solely those of the authors and do not necessarily represent those of their affiliated organizations, or those of the publisher, the editors and the reviewers. Any product that may be evaluated in this article, or claim that may be made by its manufacturer, is not guaranteed or endorsed by the publisher.
