# Supplementary material for: Significant Nutritional Gaps in Tibetan Adults Living in Agricultural Counties Along Yarlung Zangbo River
Source: Front Nutr. 2022 Apr 8;9:845026. doi: 10.3389/fnut.2022.845026 (PMC9024302; doi:10.3389/fnut.2022.845026)
Supplement: Supplementary file 1 [file Data_Sheet_1.pdf]

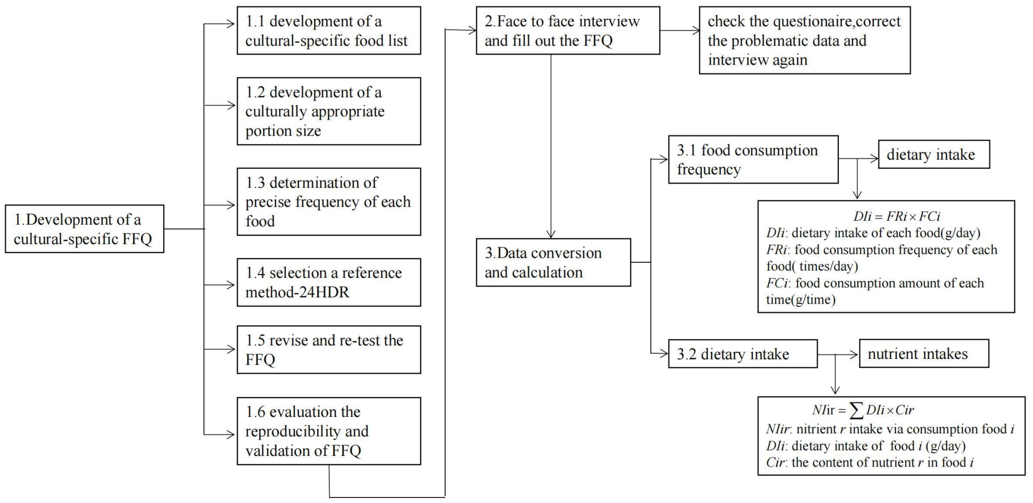

Figure S1. Methodological design for (1) the development of FFQ , (2) interview and fill out FFQ, (3) data conversion and calculation. FFQ: food frequency questionnaire; 24HDR: 24-hour dietary recall

Validated FFQs are a gold standard method of collecting information on quantity and frequency of foods consumed retrospectively. We conducted a pre-survey to develop a draft food list in order to complete a cultural-specific FFQ. Considering the relative singularity of dietary structure and ethnic uniqueness of Tibetan local residents, we first verified the validity of the FFQ by using 24-hour dietary record in Lhundup County before being applied in the present study. The FFQ was revised twice by local nutritional staff, and its re-tested results ensure that this cultural-specific FFQ can fit local daily food consumption patterns well(Table S1). The final version of the FFQ included 165 food items with average standard portion size , proper frequency categories.

To validate the FFQ for this study, a 2-consecutive-day 24 hour dietary records(24-H DR) were performed on randomly selected days in June 2020 in Lhundup County. Due to the geographical factors of the plateau and the education level of the subjects, we chose researchers familiar with the local language to track and record. We asked the subjects to record all foods in details, including seasonings and beverages, method of preparation, and to measure the weight or size of each food. We collected and carefully checked all dietary records, and average all food intakes in each 24-H DR and then considered them as the individual's habitual food intake.

Table S1. Intakes for food groups for 24 hour DR with FFQ

	**Food group intakes**		**Spearman correlation coefficients**
	**24-H DR**	**FFQ**	**24-H DR vs FFQ**
Cereals(g/day)	109±21	117±27	0.72
Vegetables(g/day)	79±11	90±19	0.55
Fruits(g/day)	82±12	97±25	0.42
Meats(g/day)	91±17	100±26	0.70
Aquatic products(g/day)	0.9±0.01	2±0.1	0.31
Eggs(g/day)	32±9	38±11	0.38
Dairy products(g/day)	98±19	114±29	0.57

Soybeans and nuts(g/day)	30±7	42±12	0.29
Oil(g/day)	26±6	20±5	0.41
Salt(g/day)	7±1.1	4±0.9	0.36
Water(ml/day)	1055±271	889±256	0.62
Median	0.48		

Food group intakes for 24-H DR and FFQ are means±SD

Spearman correlation coefficients ≥0.26（p<0.05） and ≥0.35（p<0.01）

Table S1 shows the means and correlations of the 24-H DR and the FFQ. Most of the mean crude food intakes calculated by the FFQ were systematically lower than those calculated by the 24-H DR, excluding oil and salt. Spearman correlation coefficients between the FFQ and the 24-H DR were calculated to examine the validity of the FFQ. Median and range values of Spearman correlation coefficients for crude food intakes estimated by FFQ and 24-H DR were 0.48, 0.29(soybeans and nuts)~0.72(cereals). Therefore, the FFQ developed for a Tibetan adults cohort to estimate habitual food intakes has reasonable reliability.
